# Maternal stress effects across generations in a precocial bird

**DOI:** 10.1098/rsos.231826

**Published:** 2024-08-28

**Authors:** Marion Charrier, Sophie Lumineau, Isabelle George, Maryse Meurisse, Marion Georgelin, Rupert Palme, Frédéric Angelier, Vincent Coustham, Céline Nicolle, Aline Bertin, Anne-Sophie Darmaillacq, Ludovic Dickel, Daniel Guémené, Ludovic Calandreau, Cécilia Houdelier

**Affiliations:** ^1^ Univ Rennes, CNRS, Normandie Univ, EthoS (Éthologie animale et humaine), UMR 6552, Rennes, France; ^2^ CNRS, IFCE, INRAE, Université de Tours, PRC, 37380 Nouzilly, France; ^3^ SYSAAF, Centre INRAE Val de Loire, 37380 Nouzilly, France; ^4^ Department of Biomedical Sciences, University of Veterinary Medicine, Vienna, Austria; ^5^ Centre d'Etudes Biologiques de Chizé, CNRS-LRU, UMR 7372, 79360 Villiers en Bois, France; ^6^ INRAE, Université de Tours, BOA, 37380 Nouzilly, France; ^7^ INRAE, Université de Pau et des Pays de l'Adour, E2S UPPA, NUMEA, 64310 Saint-Pée-sur-Nivelle, France; ^8^ Normandie University, UNICAEN, University of Rennes, CNRS, EthoS (Éthologie animale et humaine), UMR 6552, 14000 Caen, France

**Keywords:** maternal effects, prenatal stress, generation, offspring, emotional reactivity, Japanese quail

## Abstract

Prenatal maternal stress (PMS) is known to shape the phenotype of the first generation offspring (F1) but according to some studies, it could also shape the phenotype of the offspring of the following generations. We previously showed in the Japanese quail that PMS increased the emotional reactivity of F1 offspring in relation to (i) a variation in the levels of some histone post-translational modification (H3K27me3) in their brains and (ii) a modulation of the hormonal composition of the eggs from which they hatched. Here, we wondered whether PMS could also influence the behaviour of the second (F2) and third (F3) generation offspring due to the persistence of the specific marks we identified. Using a principal component analysis, we found that PMS influenced F2 and F3 quail profiles with subtle differences between generations. It increased F2 neophobia, F3 fearfulness and F3 neophobia but only in females. Interestingly, we did not find any variations in the level of histone post-translational modification in F3 brains and we observed inconsistent modulations of androstenedione levels in F1 and F2 eggs. Although they may vary over generations, our results demonstrate that PMS can have phenotypical effects into the third generation.

## 1. Introduction

In birds, maternal stress during egg formation (i.e. prenatal maternal stress: PMS) has long been considered an essential factor in shaping offspring phenotype. PMS can influence the morphological and physiological development of offspring [1–5] as well as a wide range of their behavioural and cognitive traits [6–10]. Interestingly, PMS effects can also influence the phenotype of the offspring further than the first generation (i.e. F1 offspring). Some studies have shown that stress experienced by laying females affects the phenotype of the second generation (i.e. F2 offspring), even when both the F1 and the F2 offspring are raised under non-stressed conditions. For instance, in zebra finches (*Taeniopygia guttata*), experimentally increasing circulating corticosterone levels during egg laying influences the weight gain of F2 offspring [11]. Additionally, in Japanese quail (*Coturnix japonica*), exposure of laying females to a stress procedure increases the emotional reactivity of the F2 offspring [12]. While the study of the F1 and the F2 offspring is essential to provide a better understanding of PMS long-term consequences, it seems interesting to go further. Indeed, the phenotypic effects observed in F1 and F2 offspring can result from direct exposure to PMS. When a F0 female is exposed to stressful conditions during her egg-laying phase, it modifies the *in ovo* environment and affects the developing F1 embryo and its germ cells, the future F2. To ensure that the phenotypic effects observed in the offspring are indirect effects of PMS, it is thus necessary to study at least the offspring of the F3 generation, since F3 offspring have never been directly exposed to F0 female experiences [13,14]. To date, studies examining the phenotypic effects of PMS beyond the F2 generation are rare. Most of them were interested in invertebrates or rodents with a focus on the effects of maternal exposure to variable diet quality or chemical compounds [15–23]. To the best of our knowledge, only one avian study has previously shown maternal effects on F3 offspring behaviour. Leroux and colleagues [24] studied the influence of genistein, an environmental contaminant, through injections into F0 Japanese quail eggs. After three generations without any additional injections, they showed that genistein treatment delayed F3 females' sexual maturity and influenced F3 offspring's body weight and reaction to social isolation [24].

The effects of PMS may involve different mechanisms. In birds, it involves at least two non-exclusive pathways. PMS effects could be mediated through epigenetic mechanisms such as DNA methylation or histone post-translational modification. These biochemical mechanisms that may influence gene expression without altering the DNA sequence [13,25] can be modulated in response to the environment [26]. As suggested in many studies, they therefore appear to be good candidates for inducing the effects of PMS on the offspring phenotype [9,10,27–29]. For example, Lindqvist *et al.* [9] found that offspring of stressed white leghorn chickens (*Gallus gallus*) had a reduced spatial learning ability and presented differential brain gene expression that mirrored that of their parents. The effects of PMS may also involve yolk maternal hormones such as sexual steroids (e.g. testosterone, androstenedione and progesterone). Many studies on a large range of avian species have shown that the level of yolk sexual steroids varies according to the quality of the maternal environment and influences the offspring's phenotype [30–33]. For example, artificial or natural variations in yolk concentrations of testosterone, androstenedione or progesterone are associated with modulations of the offspring's emotional reactivity in the Japanese quail [7,27,34], the chicken [35] and the pied flycatcher (*Ficedula hypoleuca*) [36]. Yolk maternal hormones could also be involved in the mediation of PMS effects across generations. Guibert *et al.* [12] have shown in the Japanese quail that stressful conditions experienced by laying F0 females induce an increase in testosterone concentrations in the eggs of F0 and F1 females and an increase in the emotional reactivity of F1 and F2 offspring.

In a previous experiment, we studied Japanese quail, a precocial bird particularly suitable to question PMS effects across generations (e.g. highly controllable peri- and postnatal environments, possible exclusion of maternal care effects, very short generation time [37]). During their laying phase, we exposed F0 females to unpredictable stressors. We found that the stress experienced by F0 females enhanced the emotional reactivity of their F1 offspring, probably related to the modulation of testosterone levels in F0 eggs and to specific epigenetic modifications in F1 brains [27]. Indeed, we evaluated F0 egg quality and found that F0 stressed females laid eggs with lower testosterone concentrations. We also investigated two histone post-translational modifications in the brains of F1 offspring: the dimethylation of histone H3 at lysine 4 (H3K4me2, transcription activation [38]) and the trimethylation of histone H3 at lysine 27 (H3K27me3, transcription repression [38]). We chose to focus on these specific marks because of their sensitivity to acute and chronic stress [39] and their involvement in the regulation of emotional reactivity-related behaviours in rodents and humans (e.g. anxiety-like behaviour [40,41] and fear conditioning [42]). Although PMS did not affect H3K4me2 levels, we showed that it significantly increased the number of cells displaying H3K27me3 signals in the hippocampus, the paraventricular hypothalamic nucleus (PVN) and the dorsal amygdala [27], brain structures involved in the control of vertebrates' emotional responses [43–46]. Regarding these results, we wondered whether the physiological and epigenetic effects of PMS we observed could persist and cause, across more than two generations, a similar increase in emotional reactivity to that demonstrated in the F1 offspring.

Thus, in the present experiment, we investigated the long-term effects of PMS by studying F2 and F3 offspring from the F0 females mentioned above. We studied F2 and F3 offspring emotional reactivity in challenging situations and hypothesized that the behavioural effects of PMS we previously evidenced on the F1 offspring would also be observed in these subsequent generations. In a first attempt to identify the mechanisms involved in the expression of PMS effects across generations, we (i) analysed the hormonal composition of F1 and F2 eggs, from which F2 and F3 quail hatched and (ii) studied both H3K27me3 and H3K4me2 marks in regions of interest in F3 offspring brains. Similar to the F1 generation [27], we expected to observe changes in the hormonal composition of F1 and F2 eggs as well as modulations of the levels of the epigenetic marks studied in F3 offspring brains.

## 2. Methods

### 2.1. Animals and experimental design

All experiments took place in the facilities of the EthoS laboratory (Rennes, France).

In a previous study, F0 founder Japanese quail females were exposed (stressed (S), *N* = 16) or not (non-stressed (NS), *N* = 16) to a chronic stress procedure. Briefly, F0 S females were exposed during their laying period to repeated aversive events (i.e. physical restraint, food deprivation, sudden noise, etc.) four to five times a day over a period of 24 days (for more details, see [27]). This chronic stress procedure was never applied to subsequent generations (i.e. F1, F2 and F3). To produce the F1 generation, both groups of F0 females were mated with males unexposed to the stress procedure (*N* = 16) and maintained under the same living conditions as the F0 NS females. Each female was mated with two different males, and each male was mated with two F0 NS and two F0 S females. Offspring from F0 pairs constituted the F1 generation (for more details, see [27]). Once they reached sexual maturity, F1 quail were mated using a mirrored single-pair design. F1 NS females were mated with F1 NS males (*N* = 12 pairs of NS quail from different families), and F1 S females were mated with F1 S males (*N* = 11 pairs of S quail from different families). Offspring from these F1 pairs constituted the F2 generation. Once they reached sexual maturity, F2 quail were mated again using a mirrored single-pair design. F2 NS females were mated with F2 NS males (*N* = 13 pairs of NS quail from different families), and F2 S females were mated with F2 S males (*N* = 16 pairs of S quail from different families). Offspring from these F2 pairs constituted the F3 generation.

### 2.2. Analysis of eggs of F1 and F2 females

To examine the influence of PMS on egg composition, we collected eggs from F1 and F2 females (*N*_F1-NS_ = 12, *N*_F1-S_ = 10 and *N*_F2-NS_ = 12, *N*_F2-S_ = 16). We chose to collect one egg per female, as no variation in androgen deposition was observed in quail eggs over the laying sequence [47]. After their collection, eggs were stored at −20°C until analysis. Eggs and their different components (shell, albumen, yolk) were weighed, and levels of yolk steroids (testosterone, androstenedione and progesterone) were analysed at the University of Veterinary Medicine of Vienna using enzyme immunoassays (EIAs) previously described [47,48]. The intra- and interassay coefficients of variation were below 10 and 15%, respectively, for all EIAs.

### 2.3. F2 and F3 quail incubation and housing conditions

The same incubation and housing conditions were applied to each generation. F1 and F2 NS and S eggs were collected and incubated artificially (Brinsea Ova-Easy Advance 380) for 17 days [49]. For each generation, NS and S eggs were incubated in the same incubator. During the first 14 days of incubation, the eggs were maintained at 37.7°C, with a relative humidity of 45%, while automatically rotated 45° every 30 min. For the last three days of incubation, humidity was increased to 70%, and rotation was stopped to favour hatching of F2 and F3 NS and S quail. Similar hatching success was observed between the NS and S groups (F2: *N*_NS_ = 102 and *N*_S_ = 75 (102 of the 130 NS fertile eggs and 75 of the 103 S fertile eggs hatched; chi-square test, *p* = 0.316); F3: *N*_NS_ = 38 and *N*_S_ = 55 (38 of the 73 NS fertile eggs and 55 of the 89 S fertile eggs hatched; chi-square test, *p* = 0.212)). At hatching, F2 and F3 NS and S quail were identified individually by numbered and coloured leg rings. Until posthatching day (phd) 30, they were housed by eight according to their group (NS or S) in cages (100 × 70 × 62 cm^3^) each equipped with one heater (38 ± 1°C, removed on phd 15). On phd 30, their sex was determined by plumage. Then, F2 (*N*_NS-♀_ = 32, *N*_NS-♂_ = 32 and *N*_S-♀_ = 32, *N*_S-♂_ = 32) and F3 (*N*_NS-♀_ = 17, *N*_NS-♂_ = 20 and *N*_S-♀_ = 22, *N*_S-♂_ = 29) quails were newly identified using a wing band and individually rehoused in collective batteries (35 × 24.5 × 18 cm^3^). NS females and males and S females and males were placed in four different rooms according to their group and their sex. All the birds were exposed to similar conditions of photoperiod (12 h : 12 h light–dark cycle), temperature (18 ± 1°C) and humidity (40 ± 10%). They could not see each other, but as the four rooms in which they were housed were side by side, they were exposed to a homogeneous sound environment and could hear each other. The four groups were also exposed in the same way to the presence of the experimenters. When one room and thus one group was visited, the other three were also visited.

### 2.4. F2 and F3 quail growth

The body weight of F2 and F3 quail, an indicator of their somatic growth, was measured twice, at hatching and on phd 21.

### 2.5. F2 and F3 quail emotional reactivity

Emotional reactivity is defined as an individual's capacity to perceive and react to potentially anxiogenic situations [50]. As this behavioural trait is multidimensional [51,52], we characterized several of its dimensions in the F2 and F3 offspring using three behavioural tests. First, we used a tonic immobility test to evaluate quail antipredator response, a proxy of their intrinsic fearfulness [53]. Then, we used an emergence test to assess quail reactivity to social isolation as well as quail fearfulness in a novel environment [54,55]. Finally, we used a novel object test to evaluate quail neophobia [56] (for more details about the number of F2 and F3 quail observed during each test, see electronic supplementary material, table S1).

#### 2.5.1. Tonic immobility test

Each quail was placed on a U-shaped device, on its back with its head hanging down. Quail was maintained in this position for five seconds and then gently released. If the quail was still immobile after 10 s, then the experimenter recorded the total duration of immobility (including the first 10 s); if not (i.e. if it escaped), then a new induction was attempted. If tonic immobility could not be induced after five attempts, a maximum score of five was given for the number of inductions, and a score of zero was recorded for the duration (in seconds). When a quail had not stood up after five minutes, the test was stopped, and a maximum score of 300 s was recorded. A low number of inductions and a high duration of tonic immobility are associated with a high level of intrinsic fearfulness [53]. This test was conducted between 09.00 and 15.00, from phd 9 to 10 in the F2 generation and on phd 10 in the F3 generation.

#### 2.5.2. Emergence test

Each quail was placed in a dark starting box (18 × 18 × 18 cm^3^) for one minute. To evaluate the reactivity of the quail to social isolation, the experimenter recorded the latency of emission of the quail's first rally call and the number of rally calls it produced. The emission of rally calls is considered to be positively correlated with the motivation to join conspecifics in quail [57]. After one minute, the door was opened, and the quail was allowed five minutes to leave the starting box and enter into a novel, lighted device (60 × 56 × 35 cm^3^). The experimenter, not visible to the subjects, recorded the emergence latencies of the quail's whole body. These latencies are positively correlated with fear level [56]. If the quail had not left the starting box after the five-minute period, the test was stopped, and a maximum score of 300 s was recorded. When the quail entered the novel cage, the experimenter noted, during three minutes, the latency of emission of the quail's first rally call and the number of calls produced. The experimenter also noted quail's occurrences of fear behaviours (runs, jumps, moving away, freezing, feather ruffling, low and high observations, and fear postures), which are positively correlated with their level of fearfulness. This test was conducted between 08.00 and 18.00, from phd 15 to 19 in the F2 generation and from phd 16 to 17 in the F3 generation.

#### 2.5.3. Novel object test

The day prior to the test, each individual home cage was equipped with an opaque partition to prevent the quail in the same room from seeing the novel object before being tested. The novel object was an unfamiliar terracotta cup (6 × 7 cm^2^), which was placed at the entrance of the quail home cage. During a six-minute period, the experimenter visible from the quail and placed 1.50 m from the cages, noted latencies to approach and explore the novel object, which are positively correlated with a high level of neophobia. The experimenter also noted the quail's explorations of the novel object and the quail's occurrences of fear behaviours (runs, jumps, moving away, freezing, feather ruffling, low and high observations, fear postures), which are associated with low and high levels of neophobia, respectively. This test was conducted between 08.00 and 18.00, from phd 38 to 43 in the F2 generation and from phd 36 to 37 in the F3 generation.

### 2.6. Immunochemistry of H3K27me3 and H3K4me2 in F3 females’ brains

To examine the influence of PMS on brain epigenetic mechanisms, we assessed by immunohistochemistry the levels of H3K27me3 and H3K4me2 signals. We chose to focus on these specific marks because of their sensitivity to acute and chronic stress [39] and their involvement in the regulation of behaviours related to the emotional reactivity, a behavioural trait we were interested in [40–42]. H3K27 is generally implicated in silencing chromatin whereas H3K4 is generally involved in active chromatin [38]. Increased density of H3K27me3- or H3K4me2-positive cells could therefore induce the silencing or the activation of genes involved in quail emotional reactivity respectively.

We focused H3K27me3 and H3K4me2 analysis on the F3 generation because the main objective of our study was to investigate the indirect effects of PMS. Furthermore, due to our behavioural results, we studied females only. F3 females (phd 190, *N*_NS_ = 9, *N*_S_ = 9) were randomly selected within the different families studied, independent of their behavioural results.

Selected F3 females were lethally anaesthetized by intraperitoneal injection of pentobarbital, and their brains were removed and frozen until immunohistochemistry. The immunohistochemistry was performed using a rabbit anti-H3K27me3 (Millipore, Molsheim, France) or a rabbit anti-H3K4me2 (Abcam, Cambridge, UK) antibody and then a donkey anti-rabbit-488 antibody (Jackson Immunoresearch, Peterborough, UK). Once immunochemistry was performed, H3K27me3- and H3K4me2-positive cells were counted in the paraventricular nucleus of the hypothalamus (PVN), the hippocampus (Hp) and in the following subnuclei of the arcopallium/amygdala: nucleus taeniae (TnA), intermediate (IA), medial (MA), dorsal (DA) and posterior amygdaloid (PoA). All of these brain structures were chosen because of their involvement in the control of vertebrates' emotional responses [43–46]. The brain regions were identified in accordance with the stereotaxic atlas of the chick brain [58], its revised nomenclature [59] and the quail brain atlas [60]. For each quail and each brain region studied, 1 to 8 sections were analysed, depending on the extent of the region. The number of H3K27me3- and H3K4me2-positive cells was averaged (total number of positive cells divided by the total area of the counted region) to obtain a single density value per brain region (cells µm^−2^). Due to some problems with brain sections and immunohistochemistry, brain structures of interest could not be analysed in all the females studied (table 1; for more details about the protocol, see also the electronic supplementary material).

**Table 1.** Number of F3 female brains analysed, density (cells µm^−^²) of H3K27me3- and H3K4me2-positive cells (mean ± s.e.) and results of the Fisher–Pitman permutation tests. Densities are given for the paraventricular hypothalamic nucleus (PVN), the hippocampus (Hp) and subnuclei of the arcopallium/amygdala: nucleus taeniae (TnA), intermediate (IA), medial (MA), dorsal (DA) and posterior amygdaloid (PoA). NS = non-stressed; S = stressed.

	*N*_NS_–*N*_S_	NS	S	*Z*	*p*-value
H3K27me3					
PVN	6–9	4479.9 ± 346.2	4302. 7 ± 157.4	0.122	0.925
Hp	7–9	2503.4 ± 162.7	2414.0 ± 157.4	0.131	0.902
TnA	4–4	4590.3 ± 606.1	4761.5 ± 730.5	−0.194	0.771
IA	4–4	2332.5 ± 440.5	2457.2 ± 359.6	−0.236	0.829
MA	4–4	2442.4 ± 463.7	2833.4 ± 398.2	−0.669	0.514
DA	4–4	2085.5 ± 363.9	2391.0 ± 420.7	−0.579	0.629
PoA	2–3	4140.5 ± 801.5	3279.1 ± 393.5	1.071	0.400
H3K4me2					
PVN	6–8	4383.4 ± 296.6	3930.1 ± 198.7	1.338	0.188
Hp	6–8	3231.2 ± 278.4	2951.0 ± 91.25	1.145	0.292
TnA	5–8	5136.0 ± 277.9	4060.0 ± 350.7	1.888	0.056
IA	6–8	3097.8 ± 235.8	3299.0 ± 126.9	−0.860	0.422
MA	6–8	3446.4 ± 413.2	3522.5 ± 224.0	−0.191	0.860
DA	6–8	3316.2 ± 210.1	3245.0 ± 160.1	0.297	0.777
PoA	5–7	4260.0 ± 373.5	3855.4 ± 310.0	0.848	0.409

### 2.7. Statistical analyses

Statistical analyses were computed using R v.3.6.2 software (R Core Team, 2019).

#### 2.7.1. Offspring weight

We analysed F2 and F3 quail weight with a linear mixed model (LMM; packages nlme [61] and car [62]). The fixed factors were the group (NS or S), the day (hatching, phd 21), the sex (M or F, male or female) of the individual and the interactions between group and sex and group and day. The random factor was bird identity. Model assumptions were checked by visual inspection of residual and fitted value plots (package RVAideMemoire [63]). When the residuals did not fulfil the conditions for application of the model, data were log transformed. When interactions were significant, analyses were followed by a multiple comparisons test (package emmeans [64]).

#### 2.7.2. Offspring emotional reactivity

To characterize the global emotional reactivity of quail, we performed a principal component analysis (PCA) for both the F2 and F3 generations (package FactoMineR [65]). For each PCA, we combined the raw data of the 12 behavioural variables collected during the tonic immobility, emergence and novel object tests. We used the variance inflation factor (VIF; package usdm [66]) to measure the amount of multicollinearity between all variables. For each generation, the 12 behavioural variables collected presented a VIF lower than a predefined threshold of 5 [67] so they were all included in the PCA. If missing data were present, they were automatically substituted by the mean value of both NS and S quail for the variable in question. The replacement of missing data by the mean value of the variable concerns the quail that have not emerged from the starting box during the emergence test (F2: *N*_NS_ = 1, *N*_S_ = 2; F3: *N*_NS_ = 3, *N*_S_ = 2, only for the variables recorded in the device: the latency of the rally call emission, the number of rally calls and the number of fear behaviour). It also concerns the quail that have not been tested in the novel object test (F2: *N*_NS_ = 5, *N*_S_ = 6; F3: *N*_NS_ = 1). For each PCA, we retained principal components (PCs) with eigenvalues greater than 1 [68], and we chose a criterion of factor loading of |0.5| or higher to consider that a variable was relevant to those specific PCs [68].

After completing each PCA, we extracted individuals' factorial scores for all the selected components (PCs), and we analysed them using linear models (LMs; packages nlme [61] and car [62]) to quantify the emotional reactivity of the F2 and F3 offspring. Individual factorial scores were set as a response variable, while the group (NS or S), the sex (M or F) of the individual and their interaction were set as fixed factors. Contrary to what we had planned, the family identity (F0 female identity) could not be added to the models as a random factor. Indeed, during F2 quail hatching, the separators in the hatchery fell and quail from the same group (NS or S) but from different families were mixed. It was therefore impossible to know with certainty from which family each F2 quail and so each F3 quail came. For each generation, model assumptions were checked by visual inspection of residuals and fitted value plots (package RVAideMemoire [63]). When interactions were significant, analyses were followed by a multiple comparisons test (package emmeans [64]).

#### 2.7.3. Eggs of F1 and F2 females and H3K27me3 and H3K4me2 in F3 females' brains

Data related to the F3 females’ immunochemistry and the F1 and F2 eggs were analysed using Fisher–Pitman permutation tests (package coin [69]) with 10 000 permutations. The fixed factor was the group (NS or S).

## 3. Results

### 3.1. Eggs of F1 and F2 females

We found that eggs of F1 S females contained less albumen than those of F1 NS females (table 2; Fisher–Pitman permutation, *Z* = 2.392, *p* = 0.014). Moreover, considering hormonal concentration in ng g^−1^ of yolk, F1 S yolks contained lower androstenedione concentrations than F1 NS yolks (table 2; Fisher–Pitman permutation, *Z* = 1.962, *p* = 0.048). This difference was not observed in F2 eggs (table 2; Fisher–Pitman permutation, *Z* = −1.659, *p* = 0.097). However, eggs of F2 S females contained more yolk than those of F2 NS females (table 2; Fisher–Pitman permutation, *Z* = −2.094, *p* = 0.034). Thus, considering the total amount of hormones per yolk, F2 S yolks contained higher androstenedione concentrations than F2 NS yolks (table 2; Fisher–Pitman permutation, *Z* = −2.015, *p* = 0.044). We did not find any differences for F1 and F2 eggs’ other components or for testosterone and progesterone concentrations (table 2; Fisher–Pitman permutation, *p* > 0.05).

**Table 2.** Characteristics of F1 and F2 females’ eggs (mean ± s.e.) and results of the Fisher–Pitman permutation tests. Weight and hormonal concentrations were measured for one egg per female (F1: *N*_NS_ = 12, *N*_S_ = 10; F2: *N*_NS_ = 12, *N*_S_ = 16). NS = non-stressed; S = stressed. Significant results: *p < 0.05.

	NS	S	*Z*	*p*-value
F1				
egg weight (g)	13.2 ± 0.3	12.3 ± 0.5	1.673	0.095
shell weight (g)	1.2 ± 0.0	1.2 ± 0.0	−0.536	0.607
yolk weight (g)	4.2 ± 0.1	4.4 ± 0.2	−1.076	0.292
albumen weight (g)	7.4 ± 0.2	6.2 ± 0.3	2.392	0.014*
androstenedione (ng yolk^−1^)	368.2 ± 23.3	303.8 ± 37.6	1.466	0.143
androstenedione (ng g^−1^)	87.6 ± 5.4	68.6 ± 7.4	1.962	0.048*
testosterone (ng yolk^−1^)	109.7 ± 6.5	104.8 ± 5.2	0.578	0.563
testosterone (ng g^−1^)	26.1 ± 1.5	24.0 ± 1.2	1.049	0.303
progesterone (ng yolk^−1^)	3083.6 ± 227.2	3034.2 ± 308.4	0.134	0.899
progesterone (ng g^−1^)	734.7 ± 54.9	686.7 ± 58.2	0.607	0.555
F2				
egg weight (g)	12.3 ± 0.5	12.8 ± 0.4	−0.735	0.472
shell weight (g)	1.07 ± 0.0	1.07 ± 0.0	0.092	0.930
yolk weight (g)	3.8 ± 0.1	4.3 ± 0.2	−2.094	0.034*
albumen weight (g)	6.9 ± 0.3	6.9 ± 0.2	−0.036	0.973
androstenedione (ng yolk^−1^)	354.7 ± 47.3	500.6 ± 47.0	−2.015	0.044*
androstenedione (ng g^−1^)	90.2 ± 9.2	120.1 ± 13.3	−1.659	0.097
testosterone (ng yolk^−1^)	82.4 ± 9.0	93.2 ± 6.6	−0.988	0.323
testosterone (ng g^−1^)	21.6 ± 2.0	22.0 ± 1.6	−0.131	0.898
progesterone (ng yolk^−1^)	5440.2 ± 777.4	6262.5 ± 806.1	−0.722	0.473
progesterone (ng g^−1^)	1422.9 ± 188.1	1476.1 ± 199.1	−0.192	0.851

### 3.2. F2 and F3 quail growth

We found a significant group × sex effect on the weight of F2 quail (table 3; LMM, effect of group × sex: *X*² = 6.975, *p* = 0.008; electronic supplementary material, tables S2 and S3). F2 S females were lighter than F2 NS females (table 3; *t* = 2.434; *p* = 0.016). This difference was not found between F2 NS and S males (table 3; *t* = −1.283; *p* = 0.201).

**Table 3.** F2 and F3 quail growth (mean ± s.e.). Weight (g) of F2 and F3 quail from hatching (F2: *N*_NS_ = 96, *N*_S_ = 70; F3: *N*_NS_ = 36, *N*_S_ = 52) to phd 21 (F2: *N*_NS_ = 99, *N*_S_ = 70; F3: *N*_NS_ = 36, *N*_S_ = 51). NS = non-stressed; S = stressed.

	hatching		phd 21	
	females	males	females	males
F2				
NS	9.5 ± 0.1	9.3 ± 0.1	179.3 ± 2.3	170.5 ± 1.7
S	9.1 ± 0.2	9.3 ± 0.1	174.1 ± 2.6	177.9 ± 2.9
F3				
NS	9.4 ± 0.1	9.3 ± 0.2	174.9 ± 2.6	167.8 ± 3.8
S	9.3 ± 0.1	9.6 ± 0.2	176.3 ± 3.3	163.6 ± 3.5

We did not find any significant differences in the weight of F3 NS and S quail (table 3; LMM, *p* > 0.05; electronic supplementary material, tables S2 and S3).

### 3.3. Emotional reactivity of F2 and F3 quail

**Figure 1.**
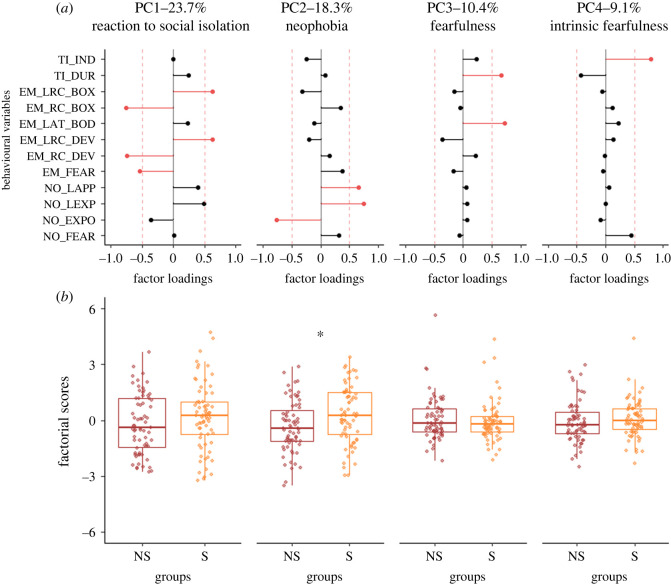
Results of the F2 quail PCA. (*a*) Factor loadings of the variables included in the PCA for each component selected (PC1, PC2, PC3 and PC4). Red bars indicate variables with significant contributions (factor loadings ≥ |0.5|). TI_IND: number of inductions during the tonic immobility test; TI_DUR: duration of immobility during the tonic immobility test; EM_LRC_BOX and EM_RC_BOX: latency to emit a rally call and number of rally calls in the starting box during the emergence test, respectively; EM_LAT_BOD: latency to quit the starting box in the emergence test; EM_LRC_DEV and EM_RC_DEV: latency to emit a rally call and number of rally calls in the device during the emergence test, respectively; EM_FEAR: occurrences of fear behaviour during the emergence test; NO_LAPP and NO_LEXP: latencies to approach and explore the cup in the novel object test, respectively; NO_EXPO: occurrences of cup observation and exploration in the novel object test; NO_FEAR: occurrences of fear behaviour in the novel object test. (*b*) Factorial scores of individuals for each component selected. For PC1, a higher score indicates a lower reaction to social isolation. For PC2 and PC3, higher scores indicate higher neophobia or fearfulness. For PC4, a higher score indicates a lower intrinsic fearfulness. Results are presented independently of the sex of the offspring (non-stressed (NS) and stressed (S)). The box plots represent the median and the first and third quartiles. The tails represent the minimum and maximum values. Dots represent individual factorial score. LM: * *p* < 0.05.

The PCA we ran to characterize F2 quails' emotional reactivity revealed four principal components explaining 61.5% of the overall variance (figure 1). The first component (PC1, 23.7% of the total variance) was represented by variables assessed during the emergence test. It differentiated individuals presenting long latencies to emit a rally call (positive loadings) from individuals producing many rally calls and fear reactions (negative loadings). As it mainly reflected the social motivation of isolated F2 quail to re-establish social contact, it was named the reaction to social isolation component. The second component (PC2, 18.3% of the overall variance) was represented by variables recorded during the novel object test. It discriminated individuals expressing long latencies to approach and explore the cup (positive loadings) from individuals expressing high occurrences of cup observations and explorations (negative loadings). As it illustrated, at least in part, F2 quails' reaction to a novel object, it was named the neophobia component. The third component (PC3, 10.4% of the overall variance) was characterized by individuals with long latencies to quit the starting box during the emergence test and long tonic immobility duration (positive loadings). As it illustrated F2 quails' reaction to a novel environment but also F2 quails' intrinsic fearfulness, it was named the fearfulness component. Finally, the fourth component (PC4, 9.1% of the overall variance) was characterized by individuals showing a high number of inductions during the tonic immobility test (positive loadings). As it mainly illustrated F2 quails' intrinsic fearfulness, it was named the intrinsic fearfulness component.

The analysis of F2 factorial scores revealed a significant group effect on the neophobia component (i.e. PC2). F2 S quail had higher factorial scores in comparison with F2 NS quail, indicating that they were more neophobic (PC2: figure 1; LM, effect of group: *F*_1,133_ = 6.680, *p* = 0.011; electronic supplementary material, tables S4 and S5). We did not find any differences between F2 NS and S quail on the components that mainly illustrate their reaction to social isolation (PC1: figure 1; LM, *p* > 0.05; electronic supplementary material, tables S4 and S5) and their fearfulness (PC3 and PC4: figure 1; LM, *p* > 0.05; electronic supplementary material, tables S4 and S5).

**Figure 2.**
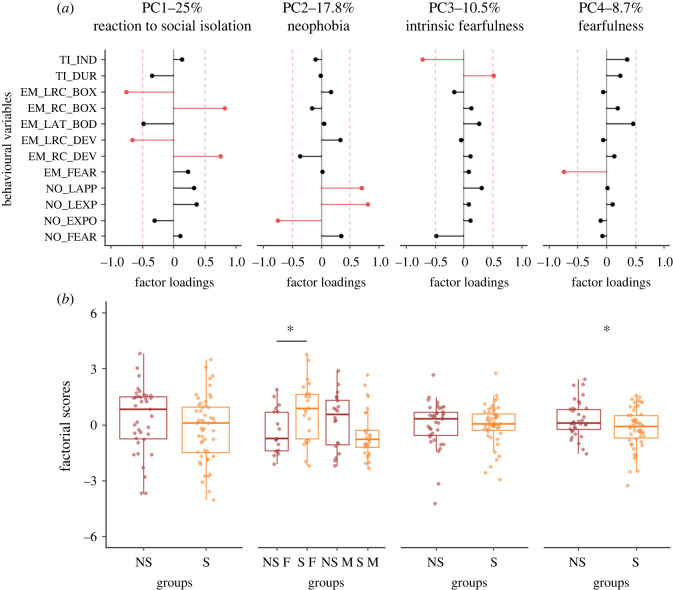
Results of the F3 quail PCA. (*a*) Factor loadings of the variables included in the PCA for each component selected (PC1, PC2, PC3 and PC4). Red bars indicate variables with significant contributions (factor loadings ≥ |0.5|). TI_IND: number of inductions during the tonic immobility test; TI_DUR: duration of immobility during the tonic immobility test; EM_LRC_BOX and EM_RC_BOX: latency to emit a rally call and number of rally calls in the starting box during the emergence test, respectively; EM_LAT_BOD: latency to quit the starting box in the emergence test; EM_LRC_DEV and EM_RC_DEV: latency to emit a rally call and number of rally calls in the device during the emergence test, respectively; EM_FEAR: occurrences of fear behaviour during the emergence test; NO_LAPP and NO_LEXP: latencies to approach and explore the cup in the novel object test, respectively; NO_EXPO: occurrences of cup observation and exploration in the novel object test; NO_FEAR: occurrences of fear behaviour in the novel object test. (*b*) Factorial scores of individuals for each component selected. For PC1, PC2 and PC3, higher scores indicate higher reaction to social isolation, higher neophobia and higher intrinsic fearfulness, respectively. For PC4, a higher score indicates a lower fearfulness. Results are presented independently of the sex of the offspring (non-stressed (NS) and stressed (S)) except if an interaction between the group and the sex was observed (non-tressed females (NS F), stressed females (S F), non-stressed males (NS M) and stressed males (S M)). The box plots represent the median and the first and third quartiles. The tails represent the minimum and maximum values. Dots represent individual factorial score. LM: * *p* < 0.05.

The PCA we ran to characterize F3 quails' emotional reactivity revealed four components explaining 62% of the overall variance (figure 2). The first component (PC1, 25% of the total variance) was represented by variables assessed during the emergence test. It differentiated individuals producing many rally calls (positive loadings) from individuals presenting high latencies to emit rally calls (negative loadings). As it mainly reflected the social motivation of isolated F3 quail to re-establish social contact, it was named the reaction to social isolation component. The second component (PC2, 17.8% of the total variance) was represented by variables assessed during the novel object test. It discriminated individuals expressing long latencies to approach and explore the cup (positive loadings) from individuals presenting high occurrences of cup observations and explorations (negative loadings). As it illustrated, at least in part, F3 quails' reaction to a novel object, it was named the neophobia component. The third component (PC3, 10.5% of the total variance) was represented by variables measured during the tonic immobility test. It differentiated individuals with long tonic immobility duration (positive loadings) from individuals showing a high number of inductions during the tonic immobility test (negative loadings). As it mainly illustrated F3 quails' intrinsic fearfulness, it was named the intrinsic fearfulness component. Finally, the fourth component (PC4, 8.7% of the overall variance) was characterized by individuals producing many fear reactions during the emergence test (negative loadings). As it could reflect both F3 quails' neophobia in a novel environment and F3 quails' general fearfulness, it was named the fearfulness component.

The analysis of F3 factorial scores revealed a significant group × sex effect on the neophobia component (i.e. PC2: LM, effect of group × sex: *F*1, 84 = 8.481, *p* = 0.005; electronic supplementary material, tables S4 and S5). Whereas F3 NS and S males had similar factorial scores (figure 2; *t* = 1.800; *p* = 0.0754), F3 S females had higher factorial scores than F3 NS females, indicating that F3 S females were more neophobic (figure 2; *t* = −2.298; *p* = 0.024). We also found a significant group effect on the component that mainly illustrate quails' fearfulness (i.e. PC4). F3 S quail had lower factorial scores than F3 NS quail, indicating that they were more fearful (PC4: figure 2; LM, effect of group: *F*_1,84_ = 4.272, *p* = 0.042; electronic supplementary material, tables S4 and S5). We did not find any differences between F3 NS and S quail on the components that mainly illustrate their reaction to social isolation (PC1: figure 2; LM, *p* > 0.05; electronic supplementary material, tables S4 and S5) and their intrinsic fearfulness (PC3: figure 2; LM, *p* > 0.05; electronic supplementary material, tables S4 and S5).

### 3.4. Density of H3K27me3 and H3K4me2 in F3 female brains

We did not find any significant differences between F3 NS and S females concerning the level of H3K27me3 and H3K4me2 in the hippocampus, the PVN and the amygdala subnuclei we studied (table 1; Fisher–Pitman permutation, *p* > 0.05).

## 4. Discussion

In a previous study on Japanese quail, we showed that PMS increases the emotional reactivity of F1 quail [27]. Here, we demonstrated that PMS effects can be observed beyond the F1 generation and influence the phenotype of unexposed offspring for at least two subsequent generations. The growth of F2 S females was delayed, and the emotional reactivity profiles of F2 and F3 NS and S quail differed. Specifically, the PCA we ran showed that F2 S quail were more neophobic than F2 NS quail. The PCA also showed that F3 S quail were more fearful than F3 NS quail and that F3 S females were more neophobic than F3 NS females. The effects of PMS we observed in this study differ subtly from one generation to the other. Indeed, the differences we found between NS and S quail in the F1 [27] generation were not found in a strictly similar way in the F2 and the F3 generations. This difference is far from surprising. The effects of PMS may have been modulated across generations by the own experiences of the F2 and the F3 quail. For example, even though we controlled for it as much as possible, the postnatal physical and social environment in which the individuals were kept may have subtly influenced F2 and F3 quail phenotypic development and therefore the effects of PMS across generations. Despite this, these results suggest that even without any direct exposure [13,14], PMS can have a long-term influence on the offspring's phenotypic development. Very few studies have explored the influence of PMS effects on the behaviour of F3 offspring in birds; information is thus extremely limited [13]. To the best of our knowledge, only one avian study has previously shown maternal effects on F3 offspring behaviour. In this study, embryos were directly exposed to signals of maternal stress through injection of genistein (an endocrine disruptor used as an environmental stressor) in F0 eggs. This has influenced F3 females' sexual maturity and F3 offspring's body weight and reaction to social isolation [24]. Our results complement this study by showing that maternal stress resulting from manipulation of the laying females' environment could also have an indirect and long-term influence on the behaviour of F3 offspring. It is very interesting to observe such effects in an oviparous model. In viviparous species, the embryo develops inside the body of the mother. The mother–embryo relationship is thus intimate and prolonged, which may result in consistent exposure to maternal effects. In oviparous species, if we exclude the incubation period as we did in our study, the mother–embryo relationship is much shorter. After oviposition, maternal effect can no longer be adjusted, which could limit their impact. Despite this, our results add to those found in insects, worms and fishes [21,70,71] and suggest that in oviparous species, as in viviparous species, maternal effects could have a strong and persistent influence on offspring behaviour. While the maternal effects we have observed are particularly interesting, it would later be relevant to consider them in the light of the specific characteristics of each family. For example, it would be interesting to develop protocols using a larger panel of individuals and a dedicated design that would enable us to examine whether one or more particular profiles of the mother as her genetic background or her personality can differentially influence the phenotype of the offspring across generations.

Interestingly, we found that PMS influenced some phenotypical traits of the F2 and F3 quail in a sex-specific manner. As mentioned above, only the weight of F2 females was influenced by PMS, and while F2 S males and females exhibited greater neophobia, these effects were only observed in females in the F3 generation. Many studies have already described such sex-specific effects within and across generations [72–77]. For instance, a maternal high-fat diet during pregnancy increased the body size of both male and female mice in the F2 generation, but these effects were evidenced only for females in the F3 generation [16,78]. Additionally, embryonic exposure of rats to vinclozolin, an endocrine disruptor, leads to a decrease in anxiety-like behaviours in F3 males but to an increase in anxiety-like behaviours in F3 females [79]. In the literature, no common pattern seems to emerge showing that one sex is more affected than another by the effects of PMS. It seems to vary according to the species studied, the stressors used or the behaviour observed [72,80–82]. However, in our study, females appeared more susceptible than males to PMS effects. The mechanisms involved here remain to be determined, but this could be explained by a different organizational effect of yolk hormones due to various hormonal sensitivities between sexes [31,83,84]. To that extent, several experiments artificially increased the yolk androgen level and found that only female growth was enhanced, whereas male growth did not change or was reduced [85,86].

One of the aims of this study was to understand whether the morphological and behavioural effects of PMS we observed in the F2 and F3 quail may be associated with the persistence of specific mechanistic marks across generations. We thus focused on two of the mechanisms that in the literature appear to be involved in the long-term effects of PMS, i.e. yolk steroid hormones and epigenetic modifications.

First, we wondered whether modulations of the yolk hormonal composition could persist across generations. We found that F1 S eggs contained lower yolk androstenedione levels than F1 NS eggs and that F2 S eggs, because of their larger yolk, contained higher yolk androstenedione levels than F2 NS eggs. Surprisingly, we observed a switch in androstenedione levels between F1 and F2 S eggs. This finding suggests that the hormonal modulations previously observed in the eggs of F0 females [27] have not been stably preserved across generations. However, we found a similar behavioural response in F2 and F3 offspring, as both F2 and F3 S quail expressed a greater emotional reactivity in comparison with F2 and F3 NS quail. Hormone-mediated maternal effects are intriguing and complex, and currently, no consensus exists regarding the effects of androgens on offspring behaviour. Some studies found that an increase in testosterone concentrations *in ovo* led to a more pronounced emotional reactivity [6,87]. Others showed the opposite and found that chicks from testosterone-injected eggs were less neophobic and fearful than the control [88]. Discrepancies among those results may be related to a multitude of factors including interactions between various hormonal and/or non-hormonal constituents [89,90] or the fact that the embryo itself could play an active role in dealing with maternal hormones, thus modulating their effects on future behaviour [90]. Another non-exclusive explanation involves the dose–response relationship of a given hormone [90–93]. Regarding our findings in F1 and F2 eggs’ hormonal composition, the relationship between androstenedione concentrations and emotional reactivity would not be linear but could rather follow a U-shaped curve with lower and higher androstenedione concentrations accentuating quail emotional reactivity. Apart from differences in their hormonal composition, we showed that eggs of F1 S females contained less albumen than those of F1 NS females and that eggs of F2 S females contained more yolk than those of F2 NS females. Albumen and yolk provide a source of essential nutrients for embryonic growth and tissue synthesis [94,95]. Previous studies showed that variations in the amount of those constituents influence offspring's morphological development [96,97] and behaviour [98]. It is therefore possible that the difference in albumen and yolk we observed in the eggs of F1 and F2 females influences the nutritional intake of F2 and F3 embryos and thus the emotional reactivity of F2 and F3 quail.

Second, we evaluated the levels of two histone post-translational modifications (i.e. H3K27me3 and H3K4me2) in the brains of F3 females. We focused this evaluation on the paraventricular hypothalamic nucleus, the hippocampus and different nuclei of the arcopallium/amygdala, brain regions known to be involved in bird neophobia and fearfulness [44,45]. We selected the K27 and K4 sites because they are associated with transcriptional repression and activation, respectively [38], and are involved in the regulation of emotional reactivity-related behaviours in mammals [40–42]. We hypothesized that changes in H3K27me3 and H3K4me2 levels could induce a large-scale reordering of chromatin, which could in turn influence the offspring phenotype and explain the behavioural effects of PMS we observed. Previously, we showed higher levels of H3K27me3 in F1 S female brains [27]. Here, contrary to what we expected, neither H3K27me3 nor H3K4me2 levels were significantly impacted in F3 offspring brains. These findings should be taken with caution due to the small size of our sample, but they seem to suggest that unlike the behavioural effects observed in the F1 generation [27], the behavioural effects observed in the F3 generation are not associated with a modulation of the levels of the epigenetic marks we studied. The apparent absence of differences in H3K27me3 and H3K4me2 levels between F3 NS and S females does not mean that epigenetic mechanisms are not involved in the effects of PMS across generations. One caveat with the approach we used is that we focused on specific marks and at the whole-cell level. As epigenetic modifications are tissue specific and sometimes even cell specific [99], it is widely possible that we missed key epigenetic modifications that could drive PMS effects across generations. As was already done in mammals, further study searching for PMS effects on a more gene-specific level would thus be highly useful to unravel the mechanisms involved here.

Given our results, the effects of PMS that we observed in the F2 and the F3 generations do not seem to be directly associated with the persistence of the hormonal and epigenetic marks we have studied. While we do not rule out the potential role of other mechanisms, it may be possible that the long-term effects of PMS we observed in the F2 and the F3 quail were generated de novo in each generation. The stress experienced by F0 females may have influenced the neural, hormonal, morphological and behavioural development of F1 quail, thus offering a different prenatal environment to their own offspring, the F2 quail. This modification of the prenatal environment could have modified the embryonic development of the F2 generation in its own way, thus inducing the phenotypic differences observed between the F2 NS and S quail. The same scenario could be envisaged to explain the phenotypic differences observed between the F3 NS and S quail. This hypothesis is supported by the results we obtained on the hormonal composition of the eggs, which varies with each generation, but also by the behavioural results, which, as we emphasized earlier, differ in subtle ways between F2 and F3 quail.

Although PMS effects vary in a dimension and sometimes in a sex-specific manner across generations, offspring emotional reactivity was generally increased in both the F2 and the F3 S quail. This result seems particularly interesting in that neither the F1, F2 nor F3 offspring were exposed to the chronic stress experienced by F0 females. We could imagine that the maintenance and magnitude of PMS effects would depend on the stability of the environment across generations. In the case of a stable environment between the F0 females and the subsequent generations, we could assume that the phenotype associated with PMS effects would be maintained or even accumulated [71,100]. When the environment between the F0 females and the subsequent generations differs, as in our study, we could imagine phenotypic adjustments or compensatory mechanisms appearing over generations [70,101]. The fact that some behavioural traits seem to be maintened, at least partially, raises questions about the adaptive potential of PMS effects. Although this question is still an ongoing debate [102,103], some studies suggest that PMS effects can be adaptive, as they can influence offspring fitness and drive their ability to cope with their subsequent environment [30,104–106]. Whether PMS effects across generations are adaptive is even more questionable. Some authors suggest that it would depend on the ability of the F0 females to predict the environmental conditions encountered by their future F2 and F3 offspring. In a predictable, relatively unchanging environment, such a prediction would be possible, and PMS effects across generations would prove adaptive. For instance, F2 waterfleas (*Daphnia cucullata*) whose F1 mothers and F0 grandmothers evolved in a similar environment (i.e. exposure to kairomones) have a larger protective helmet, giving them an adaptive advantage against predators [101]. In a complex and changing environment, however, the F0 female's ability to predict the future environment of the F2 and F3 offspring would be more difficult. Several authors claim that beyond the first generation of offspring, the adaptive potential of maternal effects would be particularly limited [21]. Some species with a short life cycle compared to potential environmental fluctuations would be an exception. For example, in a nematode (*Caenorhabditis elegans*), food deprivation in the F0 generation leads to the development of starvation resistance mechanisms in the F1 and F2 generations [20]. Determining the adaptive value of PMS across generations appears difficult in the context of our study. However, the quail has rapid development and a very short generation time [37], some common quail (*Coturnix coturnix*) born before the summer solstice in the wild could even reproduce the same year as their parents [107]. Thus, it is not unlikely that maternal effects and their influence across generations can be considered adaptive in such a species.

Here, we showed that the growth of F2 S females was delayed. Previous studies have found that high-predation environments can result in heavier offspring and earlier sexual maturity [108], suggesting that the increased mass of F2 S females could be adaptive. In addition, we did not find effects of PMS on all the dimensions of emotional reactivity that we took into account. For instance, the quail reaction to social separation did not seem to be influenced by PMS in either the F2 or the F3 generation. However, some dimensions of quail neophobia seemed to be particularly affected. As we observed in the F1 generation, PMS induced an increase in neophobia or at least a decrease in neophilia when both the F2 S quail and the F3 S females faced a novel object. This result confirms that emotional reactivity should not be considered one-dimensional. Emotional reactivity is complex and multidimensional, and to define it as well as possible, it is therefore necessary to take all its dimensions into account [51,52]. Moreover, this result suggests a strong and specific effect of PMS on offspring neophobia. Recently, neophobia has been studied in a wide range of taxa and in an evolutionary/adaptive context [109]. Generally, displaying neophobia is considered a balance encompassing the benefits to avoid unnecessary risks (e.g. encountering predators) and the costs due to missed opportunities (e.g. discovering new resources). Neophobia has been reported to be correlated with the expression of behavioural innovation [110], to play a fundamental role in the ability to cope with new environments and resources [111] and to have implications for fitness [112]. Our findings thus suggest that PMS could play a role in modulating, across generations, the capacity of birds to adapt to and survive in their living conditions.

Overall, our findings provide evidence that PMS can have important effects on the behaviour of offspring, especially on their neophobia, even into the third generation. Although further research will be necessary to identify the mechanisms that are responsible for these long-term effects, our findings suggest the possible role of PMS in modulating, across generations, birds' capacities to adapt and survive.

## Data Availability

Data and codes are available in the electronic supplementary material: ‘electronic supplementary material, _2_readme.docx’, ‘electronic supplementary material, _3_data.xlsx’, ‘electronic supplementary material_4_R-code.docx’ [113].
